# Supplementary figures and images for: In Vitro Evaluation of the Antiamoebic Activity of Kaempferol against Trophozoites of Entamoeba histolytica and in the Interactions of Amoebae with Hamster Neutrophils
Source: Int J Mol Sci. 2023 Jul 7;24(13):11216. doi: 10.3390/ijms241311216 (PMC10342687; doi:10.3390/ijms241311216)

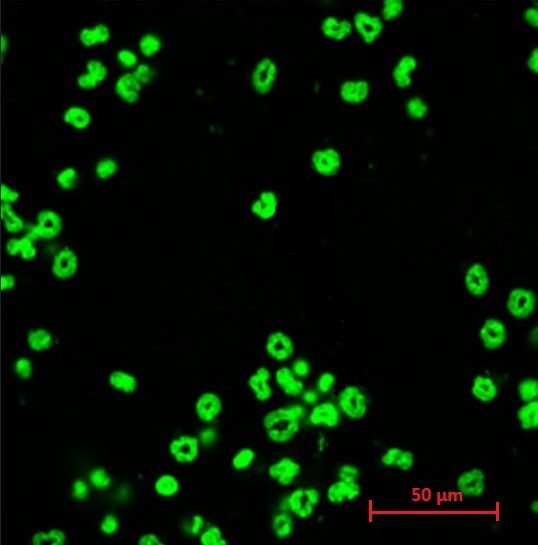

Supplement: Supplementary file 1 [file ijms-24-11216-s001.zip › ijms-2423182-supplementary.jpeg]
